# Lipophagy Impairment Is Associated With Disease Progression in NAFLD

**DOI:** 10.3389/fphys.2020.00850

**Published:** 2020-07-17

**Authors:** Simone Carotti, Katia Aquilano, Francesca Zalfa, Sergio Ruggiero, Francesco Valentini, Maria Zingariello, Maria Francesconi, Giuseppe Perrone, Francesca Alletto, Raffaele Antonelli-Incalzi, Antonio Picardi, Sergio Morini, Daniele Lettieri-Barbato, Umberto Vespasiani-Gentilucci

**Affiliations:** ^1^Laboratory of Microscopic and Ultrastructural Anatomy, University Campus Bio-Medico, Rome, Italy; ^2^Predictive Molecular Diagnostic Division, Department of Pathology, Campus Bio-Medico University Hospital, Rome, Italy; ^3^Department of Biology, University of Rome Tor Vergata, Rome, Italy; ^4^Research Unit of Pathology, University Campus Bio-Medico, Rome, Italy; ^5^Internal Medicine, Geriatrics, and Hepatology Unit, University Campus Bio-Medico, Rome, Italy; ^6^IRCCS Fondazione Santa Lucia, Rome, Italy

**Keywords:** autophagy, high-fat diet, lipolysosomes, non-alcoholic steatohepatitis, lipophagy

## Abstract

Non-alcoholic fatty liver disease (NAFLD) is the most common cause of chronic liver disease in Western countries and is associated with aging and features of metabolic syndrome. Lipotoxicity and oxidative stress are consequent to dysregulation of lipid metabolism and lipid accumulation, leading to hepatocyte injury and inflammation. Lipophagy consists in selective degradation of intracellular lipid droplets by lysosome and mounting evidence suggests that lipophagy is dysregulated in NAFLD. Here we demonstrate lipophagy impairment in experimental models of NAFLD and in a NAFLD patient cohort by histomorphological and molecular analysis. High fat diet-fed C57BL/6J male mice and high-fat/high-glucose cultured Huh7 cells showed accumulation of both p62/SQSTM1 and LC3-II protein. In 59 NAFLD patients, lipid droplet-loaded lysosomes/lipolysosomes and p62/SQSTM1 clusters correlated with NAFLD activity score (NAS) and with NAS and fibrosis stage, respectively, and levels of expression of lysosomal genes, as well as autophagy-related genes, correlated with NAS and fibrosis stage. An increased amount of lipid droplets, lipolysosomes and autophagosomes was found in subjects with NAFLD compared to healthy subjects at ultrastructural level. In conclusion, here we observed that NAFLD is characterized by histological, ultrastructural and molecular features of altered autophagy that is associated with an impaired lipid degradation. Impaired autophagy is associated with features of advanced disease. Lipopolysosomes, as individuated with light microscopy, should be further assessed as markers of disease severity in NAFLD patients.

## Introduction

Non-alcoholic fatty liver disease (NAFLD) is becoming the most common hepatic disorder in Western populations and is emerging as one of the principal causes for liver cirrhosis, hepatocellular carcinoma (HCC), and liver transplantation (Bedogni et al., 2014). NAFLD is significantly associated with conditions of hyperglycemia, dyslipidemia, obesity and other features that characterize the metabolic syndrome specially occurring in older individuals (Stefan et al., 2008). Recent studies suggest that advanced age leads to more severe histological changes and lower clinical response as hepatic lipids accumulate over time (Mofrad et al., 2003; Angulo, 2010; Bouchi et al., 2016; Hashimoto et al., 2017; Jinjuvadia et al., 2017; Leung et al., 2017; Sookoian and Pirola, 2017). Indeed, the main feature of NAFLD is steatosis, which consists in the overload of intracellular neutral lipids in the form of lipid droplets (LDs) due lipid homeostasis disruption (Gluchowski et al., 2017). In particular, the uptake and synthesis of lipids overwhelm the ability of the cell to degrade or secrete lipids. The majority of NAFLD patients have simple steatosis or steatosis with non-specific changes and do not progress or evolve very slowly to advanced liver disease (Vernon et al., 2011). Conversely, a subgroup of patients with NAFLD presents the histological criteria for non-alcoholic steatohepatitis (NASH) and has a significant risk of progression toward cirrhosis and HCC (Vernon et al., 2011).

Expanding the knowledge of factors involved in the development and progression of hepatic steatosis and NAFLD is mandatory in order to enhance the repertoire of tools for assessing disease severity and optimizing treatment. LD autophagy, the so-called lipophagy, is the main process responsible for lipid catabolism in the liver. It is a finely regulated step mechanism that consists in: (1) protein-mediated sequestration of cargo (portion or whole LD) within cytosolic double-membrane vesicles, and consequent formation of a phagosome; (2) tracking and transport of autophagosome to lysosome; (3) fusion between autophagosome and lysosome and formation of the autophagolysosome; (4) cargo degradation by lysosomal lipases.

In the early stage of the process, i.e., during the formation of autophagosomal membranes, cytosolic LC3 (LC3-I) is conjugated to phosphatidylethanolamine (PE) by the action of two consecutive ubiquitylation-like reactions catalyzed by the E1-like enzyme Atg7 and the E2-like enzyme Atg3, in order to form LC3-II. When autophagosomes fuse with lysosomes, LC3-II is degraded by lysosomal proteases. Therefore, the increase of LC3-II level is connected with its impaired turnover and is used as a marker of reduced autophagy (Klionsky et al., 2016). p62/sequestosome (SQSTM)1 is recruited as an autophagy receptor required for selective macroautophagy connecting polyubiquitinated cargo with autophagosomes. Consequently, p62/SQSTM1 accumulation represents another hallmark of autophagy blockage.

In the last years, an increasing number of studies have shown that dysregulation of lipophagy plays a pathogenetic role in the development of NAFLD, and that all the steps of this process can be affected (Patrick and Lake, 1969; Kwanten et al., 2014; Martinez-Lopez and Singh, 2015; Schulze et al., 2017). Notably, a previous study analyzed the accumulation of p62/SQTSM1 as marker of autophagy blockage in hepatocytes of NAFLD patients (Fukushima et al., 2018). Finally, lysosomes are crucial organelles for the final phase of fat disposal, and lipolysosomes represent LD-loaded lysosomes in which lipid catabolism takes place thanks to specific hydrolases. They can be individuated as vacuoles surrounded by lysosomal related-proteins, constituting an electron dense membrane at the ultrastructural analysis (Hayashi et al., 1977, 1983; Iancu et al., 2013).

In the present study, we have assessed autophagy both in *in vitro* and in *in vivo* models of NAFLD as well as in the liver of NAFLD patients. The results presented give effort to the notion that lipophagy/autophagy is impaired in NAFLD to an extent that is tightly correlated with disease severity and progression (Berson and Pessayre, 1997; Tandra et al., 2011).

## Materials and Methods

### Patients

Fifty-nine patients who had undergone liver biopsy for suspected NASH at the Hepatology Unit of the University Hospital Campus Bio-Medico of Rome and whose paraffin-embedded liver tissue was available for further analysis, were included in the study. It was confirmed that there was no history of alcoholic intake >20 gr/day if woman and >30 gr/day if man or of use of drugs known to induce liver damage, and that the following results had been obtained: negative anti-HCV antibodies and HBsAg; antinuclear antibodies (ANA) < 1:80 and negative anti-mitochondrial (AMA), anti-smooth muscle (ASMA) and anti-liver and kidney microsomal (anti-LKM) antibodies; normal transferrin saturation and serum levels of ceruloplasmin and alpha-1 antitrypsin. All patients had signed an informed consent in which the possible use of part of their hepatic tissue for research purposes was specified. The protocol of the study conformed to the ethical guidelines of the 1975 Declaration of Helsinki and was approved by the Ethics Committee of the University Campus Bio-Medico of Rome.

Liver biopsies from ten patients with normal transaminases, negative for HBsAg and anti-HCV antibodies tests, and presenting histologically normal liver with less than 5% steatosis were included in the study as control group for histological and immunohistochemical evaluations. These samples were from patients who had undergone liver surgery for metastatic tumors (liver tissue for immunohistochemistry was at least 0.5 cm from the metastatic lesion).

### Animal Model, Treatment and Biochemical Analyses

Mouse experimentation was conducted in accordance with accepted standard of humane animal care after the approval by relevant local (Institutional Animal Care and Use Committee, Tor Vergata University) and national (Ministry of Health) committees. Adult C57BL/6J (3 months-age-old) male mice (purchased from ENVIGO, Italy) were randomly divided into four groups: mice fed with normal diet (ND: 3.85 kcal/g among which 10% kcal from fat, 20% from protein and 70% from carbohydrate) for 4 months (group 1) or for 8 months (group 2); mice fed with high fat diet (HFD: 5.24 kcal/g among which 60% kcal from fat, 20% from protein, and 20% from carbohydrate) for 4 months (group 3) or for 8 months (group 4). ND (#D12450B) and HFD (#D12492) were from Research Diets, INC (New Brunswick, NJ, United States). ND contained 38% sugars, 4% saturated fats, 6% unsaturated fats and casein; HFD contained 20% sugars, 54% saturated fats, 6% unsaturated fats and casein. Mice were maintained at 23.0 ± 1.0°C and 55.0 ± 5.0% relative humidity under a 12/12 h light/dark cycle (lights on at 6:00 AM, lights off at 6:00 PM). At the end of treatment, mice were starved for 6 h before blood samples collections and body weight measurement; successively, mice were sacrificed by cervical dislocation, and liver tissues were explanted for the analyses. Blood glucose, cholesterol, alanine transaminase (ALT), aspartate transaminase (AST), and triglycerides were measured through the automatized KeyLab analyser (BPCBioSed, Italy) using specific colorimetric assay kits (BPCBioSed).

### Cells and Treatments

The hepatocyte-derived human carcinoma cell line Huh7 was obtained from the American Type Culture Collection (Rockville, MD, United States) and cultured in low-glucose Dulbecco’s Modified Eagle’s Medium (DMEM) with 10% fetal bovine serum, 1% penicillin-streptomycin, and 1% L-glutamine in a humidified incubator at 37°C with 5% CO_2_. For treatments, cells were plated at 80% confluence and, after 24 h, culture medium was replaced with complete high-glucose DMEM and 400 mM of BSA-conjugated palmitic acid was added for 72 and 96 h.

### Western Blot

Animal liver tissue was homogenized in RIPA buffer (50 mM Tris–HCl, pH 8.0, 150 mM NaCl, 12 mM deoxycholic acid, 0.5% Nonidet P-40, and protease and phosphatase inhibitors). Ten μg proteins were loaded on SDS-PAGE and subjected to Western blotting. Nitrocellulose membranes were incubated with anti-heat shock protein-60 (Hsp60) (Abcam, ab46798), anti-p62/SQSTM1 (Abcam, Ab91526), anti-LC3 (Sigma-Aldrich, L7543) primary antibodies at 1:1000 dilution. Successively, membranes were incubated with the appropriate horseradish peroxidase-conjugated secondary antibodies. Immunoreactive bands were detected by a FluorChem FC3 System (Protein-Simple, San Jose, CA, United States) after incubation of the membranes with ECL Selected Western Blotting Detection Reagent (GE Healthcare, Pittsburgh, PA, United States).

### Liver Pathology

For both human and animal liver tissue, tissue sections were stained with haematoxylin and eosin and Sirius red and each case was assessed by a single operator. In patients, the severity of steatosis (0–3), features of NASH, including necroinflammation (0–3), hepatocellular ballooning (0–2), and fibrosis stage (0–4) were assessed according to the NAFLD clinical research network scoring system (Kleiner et al., 2005), and each case was diagnosed as simple steatosis or NASH according to Brunt et al. (1999).

### Immunohistochemistry and Immunofluorescence

Each case was analyzed by immunohistochemistry for LAMP1, a lysosomal marker (ab24170, 1:50 titre, rabbit polyclonal, Abcam, Cambridge, United Kingdom) and for p62/SQSTM1 (ab56416, 1:100 titre, mouse monoclonal, Cambridge, United Kingdom), a marker of autophagic impairment. Immunohistochemistry was performed on sections obtained from formalin-fixed tissues embedded in paraffin. Immunohistochemical stained sections were scanned using the Hamamatsu NanoZoomer 2.0-RS.

LAMP1 was used to visualize vesicles of lysosomal origin (with a LAMP1-positive membrane). The number of large LAMP1-positive vesicles, with a visible lumen, i.e., lipolysosomes, was evaluated in five fields randomly chosen for each case with a magnification of 400×. A mean value was therefore derived in each sample for statistical analysis.

For the evaluation of p62/SQSTM1 expression, five fields randomly chosen were analyzed (magnification ×200) for each sample. The immunostaining was evaluated by using an open source plugin IHC Profiler for Image J (Varghese et al., 2014). All images analyzed were thresholded at the same intensity value. A scale that ranges from 0 to 3 was applied to describe the intensity of staining in each field, where 0 means negative and 3 strongly positive. The percentage of liver tissue with different score of intensity were recorded for each field and an immunohistochemistry score was derived as follows: [(3+%tissue) X3 + (2+%tissue) X2 + (1+%tissue) X1]. A mean value was then calculated for each sample (Potts et al., 2012).

Immunofluorescent staining was performed on sections from paraffin-embedded tissue and cell culture with the same antibodies used for immunohistochemistry. Secondary Alexa Fluor 488 and/or Alexa Fluor 568-conjugated donkey anti-rabbit, anti-mouse, anti-goat antibodies (Invitrogen, Carlsbad, CA, United States) were added for 30 min (1:200 titre), followed by another extensive washing step in TBS. Nuclear counterstaining was performed using Vectashield mounting medium (Vector Laboratories, Burlingame, CA, United States) containing 4,6-diamidino-2-phenylindole (DAPI). Fluorescence images were collected with a Nikon A1 Confocal Laser Microscope System (Nikon, Tokyo, Japan). Acquisition was carried out using the Imaging Software NIS-Elements (Nikon).

### Ultrastructural Analysis

A subgroup (*n* = 5) of liver samples were fixed in 2.5% glutaraldehyde in 0.1 M cacodylate buffer for the ultrastructural study. After fixation, dehydration and impregnation, the samples were included in epoxy resins and acrylic, cut at the ultramicrotome and processed for the ultrastructural study of hepatocytes by electron microscopy. In particular, morphological aspects typical of lipid overload, lysosomal activation and lipophagy/autophagy process, such as the presence of lipid droplets, membrane-bound lipid vacuoles (lipolysosomes) and autophagosomes were evaluated in 5000× fields. The presence of lipolysosomes in hepatocytes of NAFLD patients by both light and electron microscopy was evaluated according to previous criteria by Iancu et al. (2013).

### mRNA Analysis

For RT-qPCR analysis, total RNA was extracted by using Quick-RNA extraction kit (Zymo Research). RNA (3 μg) was retro-transcribed by using M-MLV (Promega, Madison, WI). qPCR was performed in triplicate by using validated qPCR primers (BLAST), Ex TAq qPCR Premix and the Real-Time PCR (Applied Biosystem). mRNA levels were normalized to Alpha-fetoprotein mRNA, and the relative mRNA levels were determined through the 2^–ΔΔCt^ method. Primers used for RT-qPCR are listed in Table 1.

**TABLE 1:** Primer sequences used for RT-qPCR analyses.

Gene	Primers
*Col1a1*	fw: CGATGGATTCCCGTTCGAGT
	rv: GAGGCCTCGGTGGACATTAG
*Col3a1*	fw: GACCTAAGGGCGAAGATGGC
	rv: AAGCCACTAGGACCCCTTTCT
*IL1B*	fw: TTCGAGGCACAAGGCACAA
	rv: TGGCTGCTTCAGACACTTGAG
*IL1b*	fw: GCACTGGGTGGAATGAGACT
	rv: GGACATCTCCCACGTCAATCT
*IL6*	fw: GAACTCCTTCTCCACAAACATGTAA
	rv: TTGTTTTCTGCCAGTGCCTCT
*Il6*	fw: GGATACCACTCCCAACAGACC
	rv: GCCATTGCACAACTCTTTTCTCA
*Mmp2*	fw. TCTGGTGCTCCACCACATAC
	rv: CCATGGTAAACAAGGCTTCATGG
*Mmp9*	fw: TGGTCTTCCCCAAAGACCTG
	rv: AGCGGTACAAGTATGCCTCTG
*TNFA*	fw: GCCCATGTTGTAGCAAACCC
	rv: TATCTCTCAGCTCCACGCCA
*Tnfa*	fw: ATGGCCTCCCTCTCATCAGT
	rv: CTTGGTGGTTTGCTACGACG

Nanostring nCounter analysis was performed in a subgroup of patients and controls (*n* = 38). Total RNA was extracted using the High Pure FFPET RNA Isolation Kit (Roche) from 10 μm sections of FFPE liver biopsies of NAFLD patients (27 specimens) and 11 controls. The RNA was quantified with the BioPhotometer^®^ D30 (Eppendorf) and 100 ng of total RNA was subjected to Nanostring nCounter Analysis System. This system utilizes a novel digital color-coded barcode technology that is based on direct multiplexed measurement of gene expression and offers high levels of precision and sensitivity (<1 copy per cell). The technology uses molecular “barcodes” and single molecule imaging to detect and count hundreds of unique transcripts in a single reaction.

We used a custom nCounter CodeSet containing 20 genes, selected in order to evaluate the expression levels (mRNA levels) of genes involved in autophagy and of other functional genes in NAFLD versus healthy liver samples. In particular, of these genes, 6 are lysosome related genes (LAMP1, LAMP2, CTSD, NPC1, ATP6V1B2, TFEB) and 9 are key genes involved in autophagy (mTOR, ULK1, BECN1, ATG12, ATG5, ATG7, p62/SQSTM1, RAB7A, GABARAP) (Table 2). We have also included in the panel 3 hepatocyte markers (AFP, ALB, and TTR) to normalize the values for the number of hepatocytes present in each sample and 2 housekeeping genes (TUBB and HIST1H3A), to normalize the values for total number of cells. Nanostring nCounter analysis was performed following the manufacturer’s protocol. In brief, 100 ng of total RNA was hybridized in solution at 65°C for 16 h with specific pairs of ∼50 base probes for each gene set mRNA. The Reporter Probe carries the signal; the Capture Probe allows the complex to be immobilized for data collection. After hybridization, the probe excess was removed and the probe/target complexes aligned and immobilized in the nCounter Cartridge. Sample Cartridges were placed in the Digital Analyzer for data collection. Color codes on the surface of the cartridge will be counted and tabulated for each target molecule. Raw counts for each mRNA was normalized (for hepatocyte markers or for housekeeping genes) and analyzed using nSolver Analysis Software (nSAS) to obtain differential expression analysis of 44 genes in NAFLD samples versus control (healthy) samples.

**TABLE 2:** Gene, Protein name and Function are derived from https://www.uniprot.org.

Gene	Protein name	Function
***Lysosomes related genes***		
LAMP1	Lysosome-associated membrane glycoprotein 1	It presents carbohydrate ligands to selectins.
LAMP2	Lysosome-associated membrane glycoprotein 2	It plays an important role in chaperone-mediated autophagy, a process that mediates lysosomal degradation of proteins in response to various stresses and as part of the normal turnover of proteins with a long biological half-life.
CTSD	Cathepsin D	Acid protease active in intracellular protein breakdown.
NPC1	NPC intracellular cholesterol transporter 1	Intracellular cholesterol transporter that acts in concert with NPC2 and plays an important role in the egress of cholesterol from the endosomal/lysosomal compartment.
ATP6V1B2	V-type proton ATPase subunit B	Non-catalytic subunit of the peripheral V1 complex of vacuolar ATPase. V-ATPase is responsible for acidifying a variety of intracellular compartments in eukaryotic cells.
TFEB	Transcription factor EB	It plays a central role in the expression of lysosomal genes. It acts as a positive regulator of autophagy by promoting expression of genes involved in autophagy.
***Autophagy related gene***		
MTOR	Serine/threonine-protein kinase mTOR	Serine/threonine protein kinase playing a central regulatory role in cellular metabolism, growth and survival in response to hormones, growth factors, nutrients, energy and stress signals.
ULK1	Serine/threonine-protein kinase ULK1	Serine/threonine-protein kinase involved in autophagy in response to starvation. It acts upstream of phosphatidylinositol 3-kinase PIK3C3 to regulate the formation of autophagophores, the precursors of autophagosomes.
BECN1	Beclin-1	It plays a central role in autophagy and acts as core subunit of the PI3K complex that mediates formation of phosphatidylinositol 3-phosphate.
ATG12	Autophagy-related protein 12	Ubiquitin-like protein involved in autophagy vesicle formation.
ATG5	Autophagy protein 5	Involved in autophagic vesicle formation.
ATG7	Autophagy-related protrein 7	E1-like activating enzyme involved in the 2 ubiquitin-like systems required for cytoplasm to vacuole transport and autophagy. It activates ATG12 for its conjugation with ATG5.
P62/SQSTM1	Sequestosome-1/Ubiquitin-binding protein p62	Autophagy receptor required for selective macroautophagy (aggrephagy). It functions as a bridge between polyubiquitinated cargo and autophagosomes. It interacts directly with both the cargo to become degraded and an autophagy modifier of the MAP1 LC3 family.
RAB7A	Ras-related protein Rab-7a	Key regulator in endo-lysosomal trafficking. It plays a role in the fusion of phagosomes with lysosomes.
GABARAP	Gamma-aminobutyric acid receptor-associated protein	Ubiquitin-like modifier that plays a role in intracellular transport of GABA(A) receptors and its interaction with the cytoskeleton. Involved in apoptosis. Involved in autophagy. Whereas LC3s are involved in elongation of the phagophore membrane, the GABARAP/GATE-16 subfamily is essential for a later stage of autophagosome maturation.
***Hepatocyte markers***		
AFP	Alpha-fetoprotein	It binds copper, nickel and fatty acids as well as serum albumin, and bilirubin less well than.
ALB	Serum albumin	The main protein of plasma having a good binding capacity for water, Ca^2+^, Na^+^, K^+^, fatty acids, hormones, bilirubin and drugs.
TTR	Transthyretin	Thyroid hormone-binding protein.
***Housekeeping genes***		
TUBB	Tubulin beta chain	Tubulin is the major constituent of microtubules.
HIST1H3A	Histone H3.1	Core component of nucleosome.

### Statistics

Depending on the parametric or nonparametric distribution, variables are expressed as mean ± SD or median and 25–75% interquartile range (25–75% IR), respectively. Differences were evaluated by the Kruskal–Wallis test. Correlations were carried out by the Spearman’s rank correlation test. A *p* < 0.05 was considered statistically significant. SPSS software (version 22.00; SPSS Inc., Chicago, IL, United States) was used for statistical analyses.

## Results

### Progressive Impairment of Lipophagy in Cell and Mouse Models of Liver Steatosis

**FIGURE 1:**
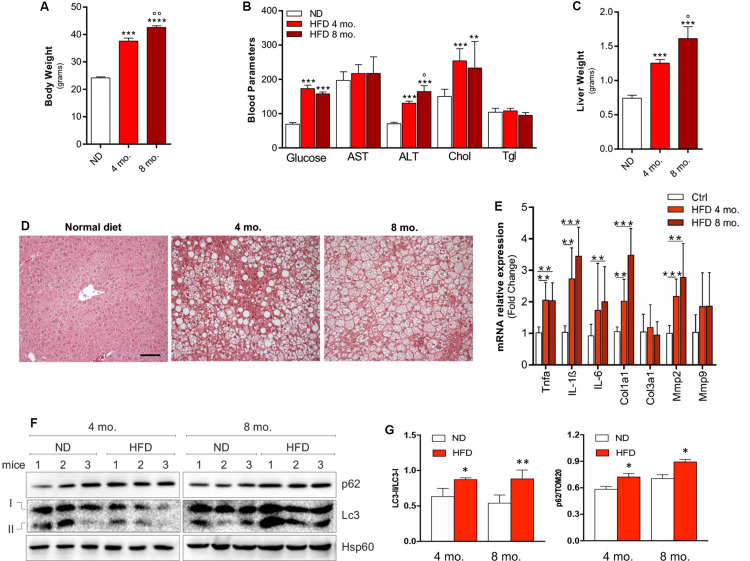
C57BL/6J male mice were fed with high fat diet (HFD) or normal diet (ND) for 4 or 8 months. Increase of body weight **(A)**, alterations of biochemical parameters **(B)**, increase of liver mass **(C)** accompanied by histological steatosis **(D)** were observed in HFD mice compared to ND. The mRNA expression of inflammation- and tissue remodeling-related genes **(E)** and autophagy protein markers LC3-II/LC3-I ratio and p62/SQSTM1 **(F)** were progressively increased as demonstrated by densitometric analyses **(G)**, indicating autophagy inhibition. HSP60 was used as loading control and representative immunoblots are reported. *p < 0.05, **p < 0.01, ***p < 0.001, ****p < 0.0001 vs. ND; °*p* < 0.05, °°*p* < 0.01 vs. 4 mo. (*n* = 6 mice per group). Original magnification: × 200. Calibration bar: 25 μm.

In order to generate a preclinical model of NAFLD, C57BL/6J mice were fed with HFD for 4 or 8 months. Mice fed with normal diet (ND) served as controls. As expected, HFD mice underwent a progressive increase of body weight (Figure 1A) and alteration of biochemical parameters such as increased blood glucose, cholesterol and ALT (Figure 1B). An increase of liver weight (Figure 1C), and the acquisition of a steatotic phenotype (Figure 1D) were also observed, in association with enhanced expression of inflammatory cytokines (IL-6, TNF-α, IL-1β) and fibrosis markers (Col1a1, Mmp2) (Figure 1E), collectively recapitulating the occurrence of a NAFLD-like clinical phenotype. At the protein level, a significant increase of both p62/SQSTM1 and LC3-II/LC3-I ratio was noticed compared to ND group that was more marked after 8 months than after 4 months of HFD treatment (Figures 1F,G).

**FIGURE 2:**
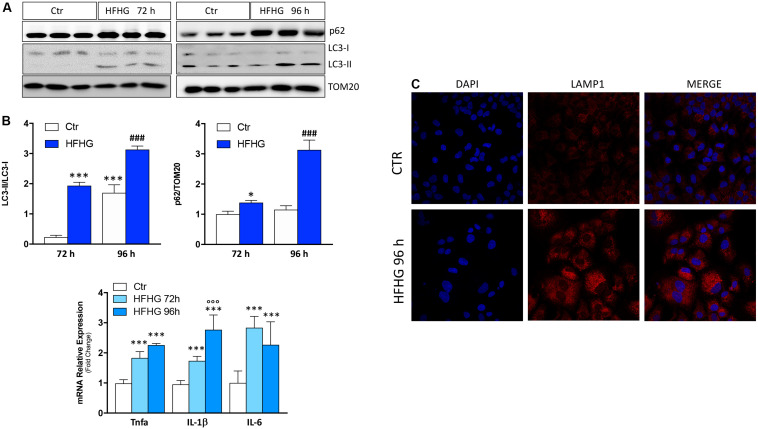
Huh7 human cells treated with 400 μM palmitate and 4.5 g/L glucose (HFHG) show accumulation of p62/SQSTM1 protein and increase of LC3-II/LC3-I protein ratio **(A)** as well as up-regulation of inflammatory cytokines **(B)** and lysosomal mass **(C)** after 72 and 96 h of treatment. TOM20 was used as loading control. Densitometric analyses of p62/SQSTM1 and LC3-II/LC3-I are shown in the bottom panel **(A)**. *n* = 3; ***p < 0.001, *p < 0.05 vs. Ctr; °°°*p* < 0.001 vs. HFHG 72 h, ^###^*p* < 0.001 vs. Ctr 96 h.

Similar results were observed in the *in vitro* model of hepatocellular fat overload. In particular, Huh7 cells were cultured in high-glucose DMEM supplemented with 400 μM palmitic acid (high-fat/high-glucose, HFHG). Starting at 72 h of treatment, up-regulation of cytokine mRNAs (IL-6, TNF-α, IL-1β), increase of both p62/SQSTM1 protein and LC3-II/LC3-I ratio (Figures 2A,B) as well as accumulation of lysosomal mass at confocal analysis with the lysosomal marker LAMP1 (Figure 2C) were observed, suggesting autophagy inhibition and lysosomal reaction.

### Increased Lipolysosomes and Impaired Lipid Catabolism in NAFLD Patients

Based on the results obtained in the experimental models of NAFLD, we moved to a cohort of 59 NAFLD patients. The main characteristics of these patients are reported in Table 3.

**TABLE 3:** Biological, anthropometric, and biochemical characteristics of the NAFLD patients.

N	59
Age (years)	49.9 ± 14.8
Sex (M/F)	35/24
BMI (kg/m^2^)	29.4 ± 4.5
AST (value/u.n.l.)	1.3 ± 0.9
ALT (value/u.n.l.)	2.2 ± 1.9
GGT (value/u.n.l.)	2.1 ± 2.0
Glycemia (mg/dL)	102.3 ± 25.3
Total cholesterol (mg/dL)	204.3 ± 48.6
HDL cholesterol (mg/dL)	47.4 ± 12.6
Triglycerides (mg/dL)	170.9 ± 117.4
Diabetes (Yes/No)	25/34
Hypertension (Yes/No)	29/30

*Data are expressed as mean ± standard deviation.*

**FIGURE 3:**
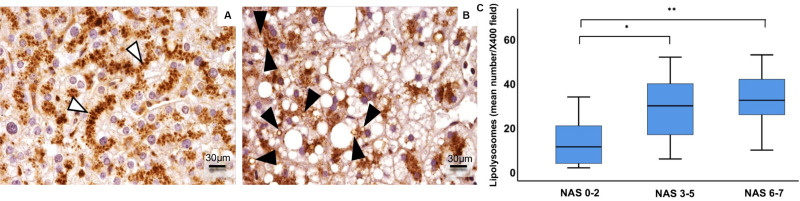
Immunohistochemical expression of LAMP1 in human hepatocyte lysosomes (LAMP1-positive particles) were regularly disposed along the path of biliary canaliculi in healthy liver **(A)**, while they were patchy distributed in fatty liver **(B)**. In fatty liver of NAFLD patients (*n* = 59), enlarged fat-loaded lysosomes, namely lipolysosomes, could be individuated as intracellular droplets with lysosomal LAMP1-positive membrane and visible lumen (**B**, arrowheads). The number of lipolysosomes/field at light microscopy increased in NAFLD patients with higher NAS (Non-alcoholic fatty liver disease Activity Score) **(C)**. Original magnification: × 400 **(A,B)**. *p < 0.05, **p < 0.01.

By immunohistochemistry for LAMP1, lysosomes from healthy subjects were detected mainly near the hepatocyte biliary pole, and no LAMP1-positive vacuoles with visible lumens were detected. In NAFLD patients, the lysosomal compartment architecture was completely disarranged. In particular, lysosomes were no longer located at the biliary pole, and LAMP1-positive vacuoles were observed inside hepatocytes. Moreover, the number of LD-loaded lysosomes, the so-called lipolysosomes, positively correlated with the NAS score (ρ = 0.375, *p* < 0.005) (Figure 3).

**FIGURE 4:**
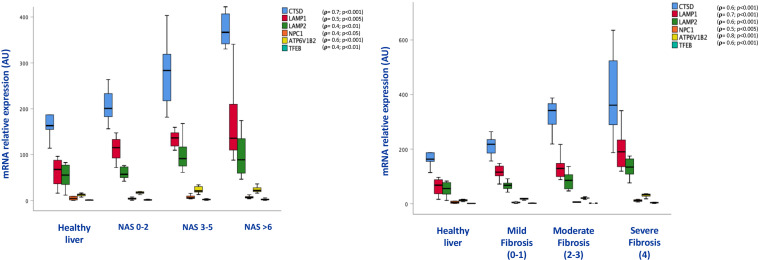
Box plots of mRNA expression of lysosome related genes in association with NAS score (on the **left**) and fibrosis stage (on the **right**); (*n* = 38). For correlation purpose Spearman’s **ρ** test was performed.

The increase of lysosomal mass was supported by increased levels of expression of lysosomal genes (CTSD, LAMP1, LAMP2, NPC1, ATP6V1B2,TFEB), whose levels of expression were correlated with the NAS score (*r* = 0.7; *p* < 0.001;*r* = 0.5; *p* < 0.005;*r* = 0.4; *p* < 0.01;*r* = 0.4; *p* < 0.05; *r* = 0.6; *p* < 0.001; *r* = 0.4; *p* < 0.01, respectively), as well as with fibrosis stage (*r* = 0.6; *p* < 0.001; *r* = 0.7; *p* < 0.00; *r* = 0.6; *p* < 0.001; *r* = 0.5; *p* < 0.005, *r* = 0.8; *p* < 0.001; *r* = 0.6; *p* < 0.001, respectively) (Figure 4).

### Impaired Autophagy in NAFLD Patients

**FIGURE 5:**
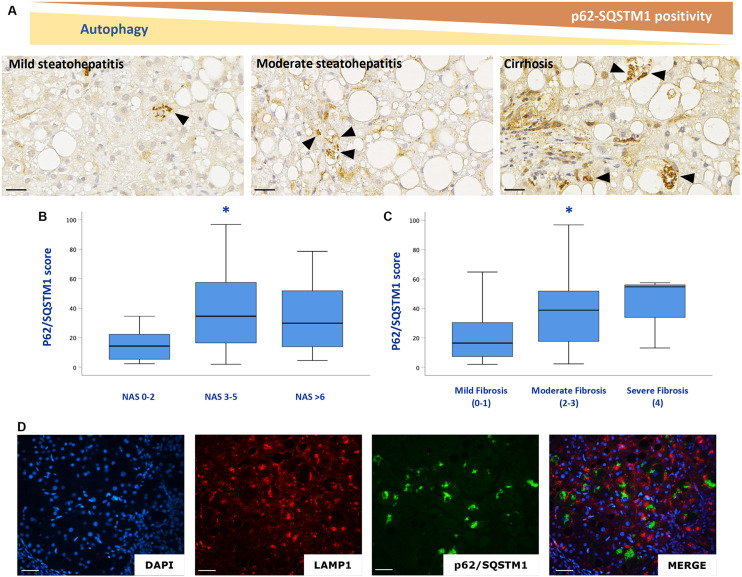
p62/SQSTM1 accumulates with NAFLD progression **(A–C)**. P62/SQSTM1 positive Mallory-Denk bodies (arrowheads) were individuated in hepatocytes. Double labeling experiments with a lysosome marker (LAMP1, red) and autophagy impairment marker (p62/SQSTM1 green), confirmed that p62/SQSTM1 accumulates outside the lysosomes **(D)**. *n* = 59. Scale bar: 25 μm; * = *p* < 0,05 vs. NAS 0–2 or mild fibrosis.

**FIGURE 6:**
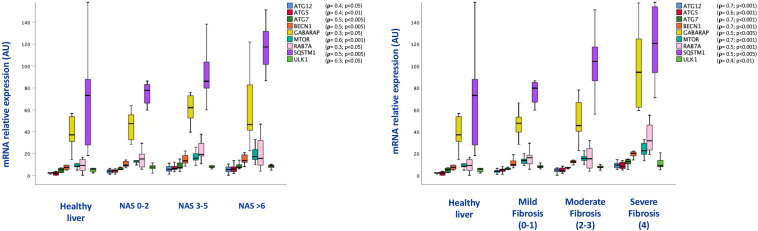
Box plots of mRNA expression of autophagy related genes in association with NAS score (on the **left**) and fibrosis stage (on the **right**); (*n* = 38). For correlation purpose Spearman’s **ρ** test was performed.

Consistent with the results obtained in mice, p62/SQSTM1 protein levels as well as fibrosis were significantly increased in the liver of NAFLD patients. In particular, immunohistochemical positivity of p62/SQSTM1 increased with progression of fibrosis, accumulating in hepatocytes in the so-called Mallory-Denk bodies, and indicating autophagy blockage. Furthermore, by means of immunofluorescence, p62/SQSTM1 aggregates were observed outside the lysosomes, suggesting and impairment also of the first steps of autophagy (Figure 5). Moreover, significant correlations between levels of expression of autophagy-related genes (ATG12, ATG5, ATG7, BECN1, GABARAP, MTOR, RAB7A, P62/SQSTM1, ULK1) and the NAS score (*r* = 0.4; *p* < 0.05; *r* = 0.4; *p* < 0.01; *r* = 0.5; *p* < 0.005; *r* = 0.5; *p* < 0.005; *r* = 0.3; *p* < 0.05; *r* = 0.6; *p* < 0.001; *r* = 0.3; *p* < 0.05; *r* = 0.5; *p* < 0.005; *r* = 0.3; *p* < 0.05, respectively) and fibrosis stage were observed (*r* = 0.7; *p* < 0.001; *r* = 0.6; *p* < 0.001; *r* = 0.7; *p* < 0.001; *r* = 0.7; *p* < 0.001; *r* = 0.5; *p* < 0.005; *r* = 0.7; *p* < 0.001; *r* = 0.5; *p* < 0.001; *r* = 0.5; *p* < 0.005; *r* = 0.4; *p* < 0.01, respectively) (Figure 6).

**FIGURE 7:**
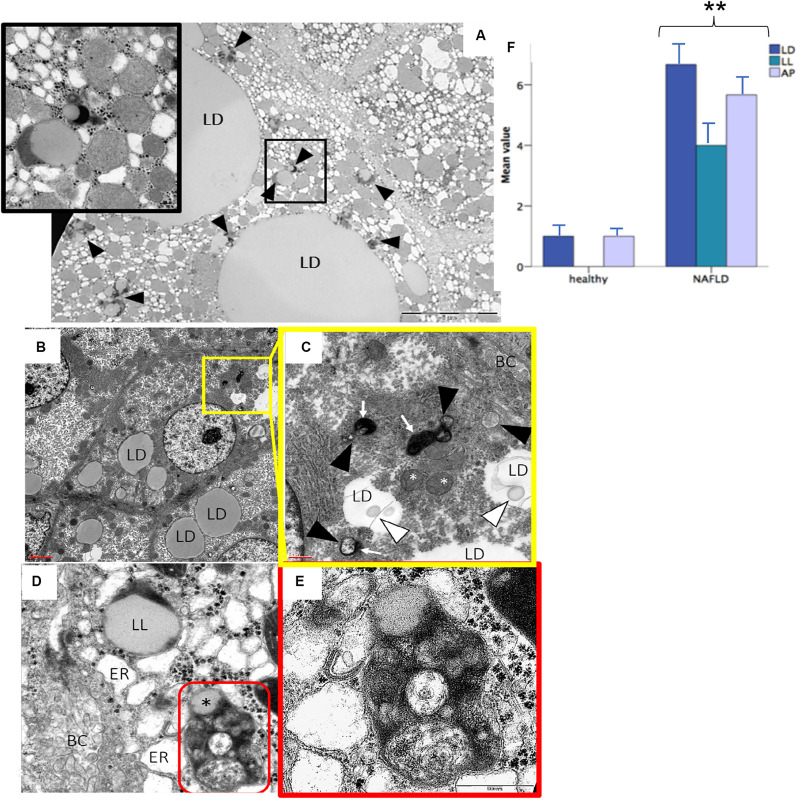
Transmission electron microscopy analysis confirmed the presence of lipolysosomes with dense lipid core and typical electron-dense lysosomal membrane in fatty liver **(A–D)**. The different cellular organelles involved in the autophagic process phases are depicted in the high power fields **(C,E)**. Bars representing the number of autophagic organelles in NAFLD patients compared to healthy subjects (mean values, *n* = 5) **(F)**. LD, lipid droplet; LL, lipolysosome; AP, autophagosome; ER, stressed endoplasmic reticulum; BC, bile canaliculus; red circle, autolysosome; white arrowhead, phagophore; black arrowhead, immature autophagosome; white arrow, lysosome; white asterisk, stressed mitochondria; black asterisk, lipid component of autolysosome. Original magnification: 3900× **(A)**; 2300× **(B)**; 12000× **(C,D)**; 46000× **(E)**. **p < 0.05.

Evaluation of autophagy was also performed at ultrastructural level in human biopsies processed for TEM experiment. An increased amount of lipid droplets, lipolysosomes and autophagosomes was found in subjects with NAFLD compared to healthy subjects (*p* < 0.05) (Figure 7).

### NAFLD Progression and Aging in NAFLD Patients

Histological markers of NAFLD progression were associated with aging in NAFLD patients. Standard histopathological features of NAFLD severity, such as grading and staging of the disease (NAS and fibrosis score, respectively) were significantly different in NAFLD patients above and below 54 years (Table 4). Furthermore, immunohistochemical positivity of p62/SQSTM1 was correlated with aging in NAFLD patients (*r* = 0.3, *p* < 0.05).

**TABLE 4:** Correlation of NAFLD characteristics with patients’ age.

Age (years)	<54	>54	Mann-Whitney U Test
Number of patients	29	30	–
NAS	2 (1.5)	2.5 (1)	*p* = 0.04
Fibrosis	1 (1)	2 (0.75)	*p* = 0.001

*Data are expressed as median (interquartile range).*

## Discussion

Aging and obesity predispose to the development of NAFLD. Actually, the chronic overload of lipids has harmful effects in the liver, exposing resident hepatocytes to their cytotoxic action (lipotoxicity) and oxidative stress, the effect of which are more pronounced with aging (Zelber-Sagi et al., 2006; Chen et al., 2013; Xu et al., 2013a). Lipophagy represents the main lipid degrading system in the liver and its impairment was recently associated with the pathogenesis of NAFLD (Sinha et al., 2014; Zhang et al., 2018). In lipophagy, the neutral lipids are partially subtracted in autophagosomes and transported to lysosomes, which represent the final destination of lipids in this pathway.

In the present study, we have demonstrated that chronic lipid overload through HFD in mice and palmitic acid treatment in a human hepatocyte derived cell line, respectively, leads to a time-dependent inhibition of autophagy. Notably, in HFD-fed mice, the progression of NAFLD-mimicking features was strongly associated with the degree of autophagy inhibition (p62/SQSTM1 and LC3-II/LC3-I increase). Moreover, here we showed, for the first time, the presence of lipolysosomes in hepatocytes of NAFLD patients by both light and electron microscopy. In NAFLD, lipolysosomes likely represent the progressive impairment of the lysosomal function, and in particular of the capability of lysosomal hydrolases to catabolize fat. This is supported by the correlation between the increased number of lipolysosomes and disease activity in terms of necroinflammation.

The up-regulation of lysosomal genes, such as CTSD, LAMP1, LAMP2, NPC1, ATP6V1B2, TFEB, suggesting an increase of the overall lysosomal mass, should be well interpreted as an attempt to counteract lysosomal dysfunction. Among these genes, notably, CTSD has been already correlated with inflammation and impairment of lipid metabolism in NAFLD. Houben et al. (2017) have shown that inhibition of CTSD in mice results in reduced cholesterol and triglyceride levels in the liver. The group of Liao showed that CTSD is up-regulated as cholesterol accumulates in lysosomal compartments in an NPC1 knock-out model, demonstrating an increase of CTSD when lysosomes are engulfed (Liao et al., 2007). In the present study, CTSD expression was higher in NAFLD patient compared to controls, and a direct correlation was observed between CTSD expression and both NAS and fibrosis.

Lysosome engulfment by lipids has been shown to inhibit autophagosome turnover, and several studies in lysosomal storage diseases have demonstrated an impairment in the fusion between autophagosomes and fatty lysosomes that leads to defective autophagic clearance (Fukuda et al., 2006; Settembre et al., 2008; Sarkar et al., 2013). Consistent with this, as already described (Fukuo et al., 2014), we confirmed an increased expression of p62/SQSTM1 mRNA and protein in the liver of NAFLD patients that was significantly associated with disease activity and fibrosis stage. The increase of p62/SQSTM1 suggests that the autophagic cargos cannot fuse with lysosomes in order to be degraded, leading to cytosolic aggregates of p62/SQSTM1; accordingly, double labeling experiment revealed that p62/SQSTM1 clusters are detached from lysosomal-derived structure. Furthermore, in NAFLD patients, TEM analysis revealed an increased number of autophagosomes and lipolysosomes, likely representing the compensatory expansion of a bad-functioning autophagic machinery. Indeed, the expression of several genes involved in autophagy (Atg7, Atg5, GABARAB, and Rab7) was significantly correlated with NAS and fibrosis stage. These are key proteins for autophagic vesicle formation and lysosome maturation, and their deficit was found to suppress autophagic flux and to impair the autophagosome-lysosome fusion (Xu et al., 2013b). Moreover, autophagy could exert an anti-fibrogenic role since it promotes survival of hepatocytes and hepatocyte apoptosis is a central event in the fibrogenic process (Ni et al., 2014; Ruart et al., 2019; Hammoutene et al., 2020).

In conclusion, in the present study, we have observed histological, ultrastructural and molecular features of altered autophagy in NAFLD, leading to impaired lipid degradation. Impaired autophagy seems to be a pivotal factor determining the clinical progression of NAFLD also considering that a decrease in autophagic activity was previously described with aging, which is associated with NASH progression (Rubinsztein et al., 2011). Individuating molecular markers of lipophagy impairment could help to identify those patients at risk of progression. In particular, in this study, LAMP1-positive vacuoles with visible lumen, known as lipolysosomes, were identified as morphological feature of altered lipophagy. Further studies are clearly awaited in order to verify if quantification of lipolysosomes could be a feasible and reproducible tool for assessing the severity of NAFLD and its propensity to progress.

## Data Availability Statement

The raw data supporting the conclusions of this article will be made available by the authors, without undue reservation, to any qualified researcher.

## Ethics Statement

The studies involving human participants were reviewed and approved by Comitato Etico dell’Università Campus Bio-Medico di Roma with authorization n° 30/15 OSS ComEt CBM. The patients/participants provided their written informed consent to participate in this study. The animal study was reviewed and approved by the University Animal Welfare Committee-OPBA, Tor Vergata University with authorization no 378/2017-PR.

## Author Contributions

SC designed and supervised the study, performed the morphological experiments and the statistical analysis, and wrote the manuscript. FZ performed molecular experiments with Nanostring and gave an intellectual contribution. SR and FV performed the immunohistochemical experiments and provided intellectual contribution. KA designed molecular experiments, provided intellectual contribution, and helped writing the manuscript. MZ contributed to the design of the electron microscopy study. MF performed fluorescence microscopy experiments. GP provided tissue samples from UCBM Hospital. FA helped in recruiting patients’ cohort from UCBM database. RA-I contributed to the critical revision of the manuscript. AP contributed to the study design and provided intellectual contribution. SM supervised the study and contributed to the critical revision of the manuscript. DL-B performed *in vivo* experiments, analyzed molecular data, and provided substantial intellectual contribution. UV-G obtained the patients informed consents, contributed substantially to the design of the study, to the discussion of the data, and to the writing of the manuscript. All authors contributed to the article and approved the submitted version.

## Conflict of Interest

The authors declare that the research was conducted in the absence of any commercial or financial relationships that could be construed as a potential conflict of interest.

## Abbreviations

AFP: alpha-fetoprotein
ALB: albumin
ATG12: ubiquitin-like protein ATG12
ATG7: ubiquitin-like modifier-activating enzyme ATG7
ATP6V1B2, v-type proton ATPase subunit B: brain isoform.
BECN1: beclin-1
CTSD: cathepsin D
GABARAP: gamma-aminobutyric acid receptor-associated protein
HFD: high-fat diet
HIST1H3A: histone cluster 1H3 family member a
LAMP1: lysosome-associated membrane glycoprotein 1
LAMP2: lysosome-associated membrane glycoprotein 2
mTOR: serine/threonine-protein kinase mTOR
NAS: NAFLD activity score
NASH: non-alcoholic steatohepatitis
NPC1: NPC intracellular cholesterol transporter 1
nSAS: nSolver Analysis Software
p62/SQSTM1: sequestosome-1
RAB7A: ras-related protein Rab-7a
TBS: tris buffered saline
TEFB: transcription factor EB
TG5: autophagy protein 5
TTR: transthyretin
TUBB: tubulin-beta
ULK1: serine/threonine-protein kinase ULK1.
